# Long-term survival following multimodal therapy for lung cancer with hepatic metastasis: a case report

**DOI:** 10.3389/fonc.2025.1663079

**Published:** 2025-10-28

**Authors:** Zhao Junjun, Zhang Jiandong, Dong Lingjun, Yu Guangmao, Zhang Chu

**Affiliations:** ^1^ Department of Thoracic Surgery, Shaoxing People’s Hospital, Shaoxing, Zhejiang, China; ^2^ School of Medicine, Shaoxing University, Shaoxing, Zhejiang, China

**Keywords:** non-small cell lung cancer(NSCLC), hepatic metastasis, surgery, radio frequency ablation(RFA), case report

## Abstract

**Background:**

The liver serves as a predominant metastatic site in advanced lung cancer, with patients developing hepatic metastases demonstrating particularly dismal prognosis and a median survival of approximately 5 months. Currently, no consensus has been established regarding optimal therapeutic strategies for lung cancer with liver metastases.

**Case presentation:**

A patient with advanced lung cancer and oligometastatic liver metastases achieved significant tumor regression in both pulmonary and hepatic lesions following combined immunotherapy and chemotherapy. Subsequent radiofrequency ablation was performed for the liver metastasis, while surgical resection of the primary lung lesion resulted in pathological complete response (pCR). The patient has maintained long-term survival without evidence of disease recurrence.

**Conclusions:**

For patients with advanced lung cancer and oligometastatic liver metastases demonstrating favorable response to combined immunotherapy and chemotherapy, consolidative surgical resection with adjunctive liver-directed local therapy may be considered, potentially conferring long-term survival benefits.

## Introduction

Lung cancer is a prevalent malignant tumor, with NSCLC being the predominant subtype. Most patients are diagnosed at advanced stages, and compared to early- or intermediate-stage cases, those with advanced disease exhibit a dismal 5-year survival rate of less than 5%, indicating poor overall prognosis. The most common metastatic sites of NSCLC include lymph nodes, bone, brain, and liver, with hepatic metastases associated with the worst outcomes—median survival is approximately 5 months (1, 2). Advanced lung cancer has traditionally been considered a contraindication for surgery, necessitating chemotherapy-based systemic therapy. However, with the success of immunotherapy and targeted treatments in advanced NSCLC, a subset of patients now achieves long-term survival. Therefore, determining the optimal treatment strategy for carefully selected NSCLC patients with oligometastatic disease (particularly isolated organ metastases) is of critical importance. This report presents a case of NSCLC with solitary liver metastasis, in which the patient attained long-term disease-free survival following combined immunotherapy and chemotherapy, pulmonary resection, and liver-directed local therapy. Our findings suggest a potential new therapeutic approach for patients with isolated hepatic metastases.

## Case presentation

**Figure 1:**
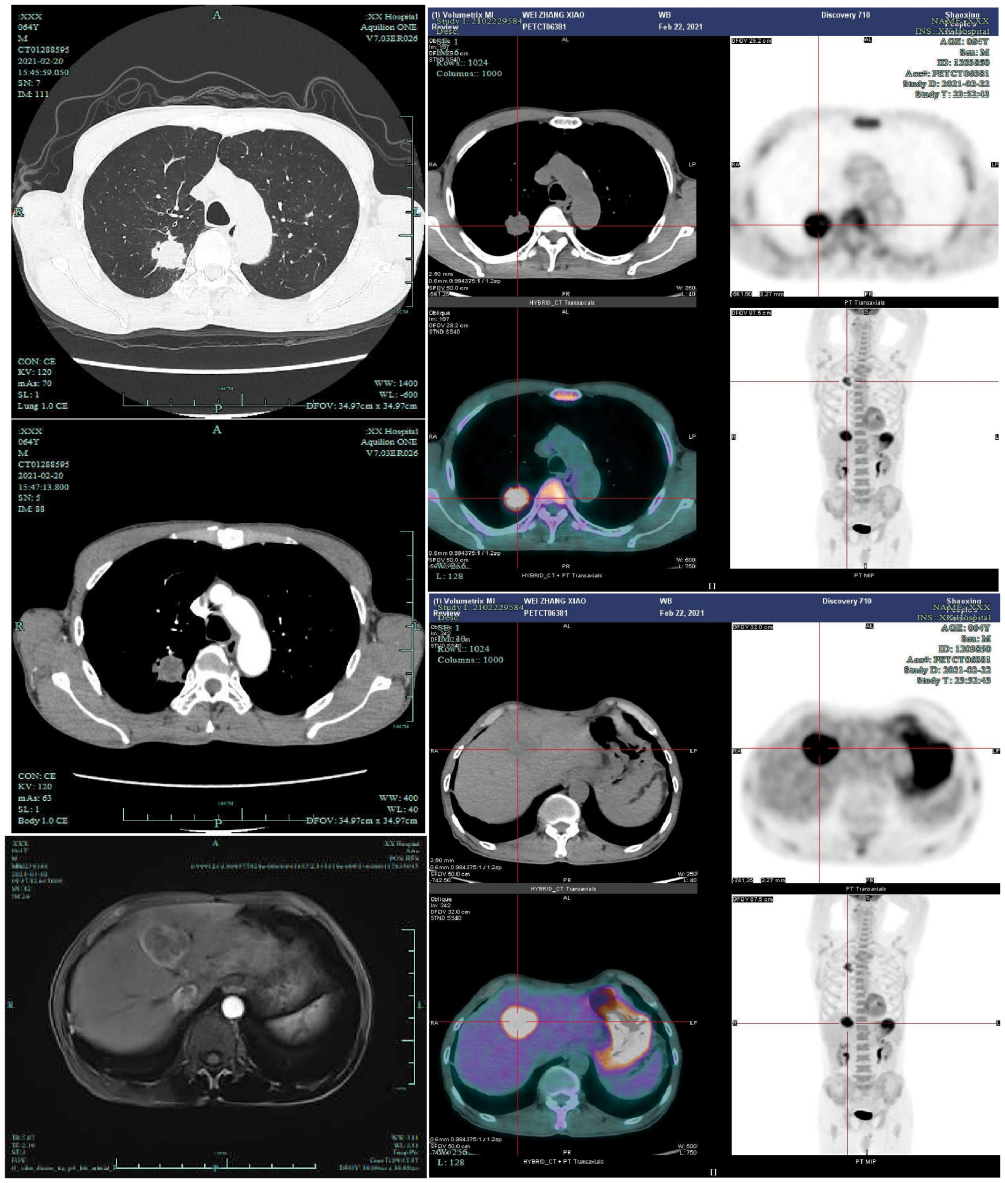
Upon admission, the patient underwent contrast-enhanced chest CT(2021-2-20), contrast-enhanced liver MRI(2021-3-2), and PET-CT examinations(2021-2-23). PET-CT demonstrated a right upper lung nodule with a maximum standardized uptake value (SUVmax) of 8.30, and a liver mass with SUVmax of 11.89 on conventional imaging and 13.00 on delayed-phase imaging.

A 64-year-old male was admitted to the Department of Respiratory Medicine at our hospital on February 20, 2021, due to the presence of a right upper lung nodule first identified more than two years earlier. The nodule was initially detected on chest CT imaging two years prior to admission and was found to have progressively enlarged during subsequent regular follow-up examinations(historical imaging unavailable due to prolonged interval). The patient denied respiratory symptoms (cough, sputum, chest pain), constitutional symptoms (low-grade fever, night sweats), or abdominal/neurological complaints. Medical history included chronic bronchitis (untreated) and a 40-pack-year smoking history (current smoker, 30 cigarettes/day), with no alcohol use or family history of malignancies. On February 20, 2021, a contrast-enhanced chest CT scan performed after admission revealed a solid nodule in the posterior segment of the right upper lobe, measuring approximately 28 × 32 mm, with a CT value of about 25 Hounsfield units. The nodule exhibited a lobulated margin with surrounding spiculation and demonstrated moderate enhancement following contrast administration. The incidental detection of a hepatic space-occupying lesion on chest CT raised suspicion of metastatic disease, prompting subsequent abdominal Magnetic Resonance Imaging (MRI) evaluation. Contrast-enhanced abdominal MRI demonstrated a 38×40 mm round lesion in segment IV of the left hepatic lobe, showing hypointensity on T1-weighted imaging and heterogeneously hyperintense signal on T2-weighted imaging, radiologically consistent with metastatic involvement. PET-CT revealed increased Fluorodeoxyglucose (FDG) uptake in both the right upper lung nodule (SUVmax=8.30) and hepatic lesion (SUVmax=11.89 on conventional imaging, increasing to 13.00 on delayed imaging) (**Figure 1**), further supporting the diagnosis of metastatic disease. Serum tumor markers including carcinoembryonic antigen (CEA) and Alpha-fetoprotein (AFP) were within normal limits, and routine blood tests, biochemical profiles, and cranial MRI showed no significant abnormalities. Pathological examination of CT-guided percutaneous needle biopsy specimens from the pulmonary nodule confirmed squamous cell carcinoma (**Supplementary Figure 1**).

The patient was definitively diagnosed with squamous cell carcinoma (SCC) of the right upper lobe with hepatic involvement. Following multidisciplinary team (MDT) evaluation, primary hepatocellular carcinoma was deemed unlikely due to:(1) Absence of hepatitis B history;(2) Normal serum AFP levels;(3) Radiologic concordance (contrast-enhanced MRI and PET-CT) supporting metastatic disease. The final diagnosis was stage IVA (cT1N0M1b) metastatic pulmonary SCC per NCCN Guidelines (Version 9) TNM staging. The MDT consensus recommended first-line chemoimmunotherapy; however, comprehensive genomic profiling was not performed due to financial constraints, despite patient consent for the proposed therapy.

The patient, with an excellent performance status (Eastern Cooperative Oncology Group Performance Status, Performance Status, ECOG PS=0), initiated first-line chemoimmunotherapy on March 9, 2021, following exclusion of contraindications. The regimen consisted of tislelizumab (anti-programmed death-1, anti-PD-1) combined with nab-paclitaxel (administered on Day 1 and Day 8) and carboplatin (AUC = 5), repeated every 21 days. Following the first treatment cycle, significant tumor regression was observed in both the right upper pulmonary nodule and hepatic lesion. Given the satisfactory therapeutic response, the original treatment regimen was maintained for consolidation therapy. During the eighth day of the third cycle (albumin-bound paclitaxel administration), treatment was interrupted due to pulmonary infection. After MDT discussion following the third cycle, the decision was made to continue the current immunotherapy combined with chemotherapy regimen, with plans for subsequent local liver-directed therapy. The patient subsequently completed two additional cycles of combined immunotherapy and chemotherapy. Ultimately, after five treatment cycles, marked tumor regression was achieved in both the right pulmonary nodule and hepatic lesion, demonstrating favorable treatment response. On October 11, 2021, the patient underwent CT-guided radiofrequency ablation for the hepatic metastasis, with an output power of 30 W and total energy delivery of 15 kJ. No post-procedural complications, such as hemorrhage or pneumothorax, were observed.

**Figure 2:**
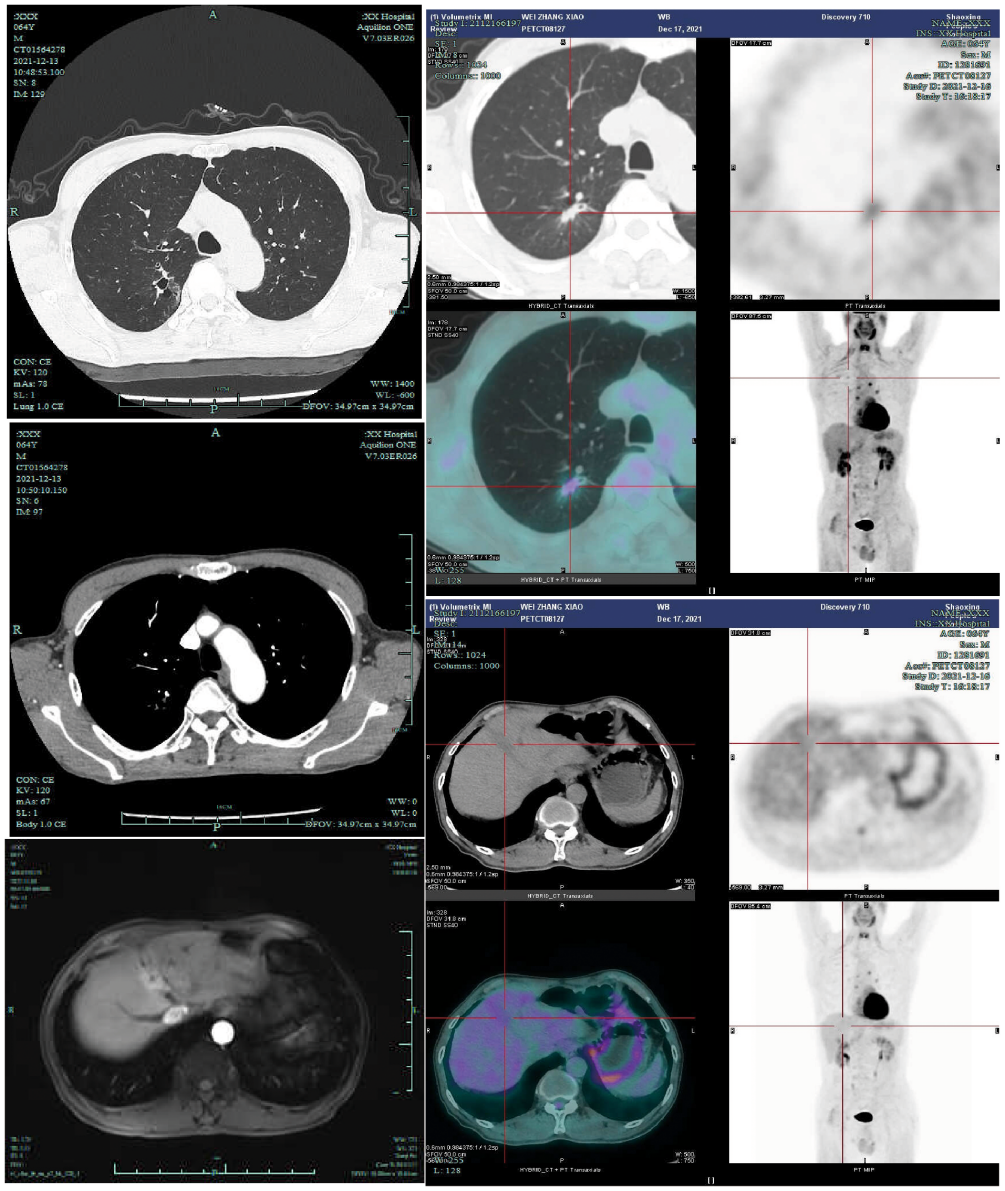
Preoperatively, the patient underwent contrast-enhanced chest CT (2021-12-13), contrast-enhanced liver MRI (2021-11-9), and PET-CT examinations (2021-12-17). Preoperative PET-CT demonstrated a right upper lung nodule with a maximum standardized uptake value (SUVmax) of 1.85, while the liver mass exhibited physiological FDG uptake.

**Figure 3:**
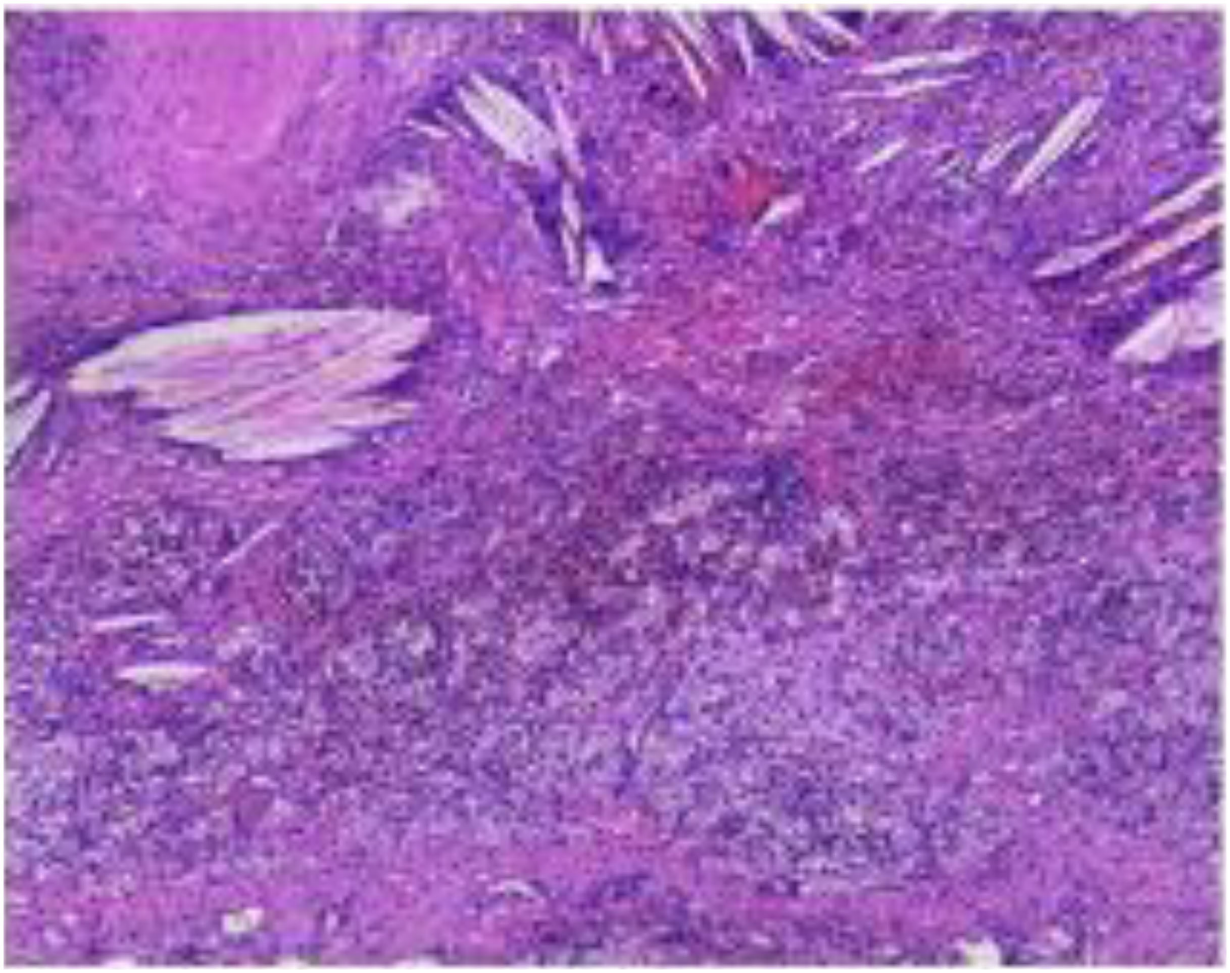
Postoperative routine histopathological sections were examined.

The patient demonstrated satisfactory systemic treatment response with complete metabolic resolution of both pulmonary and hepatic lesions on preoperative PET-CT (**Figure 2**). Following thorough MDT evaluation and considering the patient’s strong surgical preference, right upper lobectomy with mediastinal lymph node dissection was performed via video-assisted thoracic surgery on December 21, 2021. Histopathological examination revealed lymphocyte infiltration without residual tumor cells in both the resected specimen and dissected lymph nodes, confirming pCR (**Figure 3**). Postoperative immunotherapy maintenance was irregular due to COVID-19 pandemic constraints, with only two subsequent administrations of tislelizumab on March 25 and May 14, 2022. During the most recent outpatient follow-up on January 16, 2025 (**Supplementary Figure 2**) and subsequent telephone contact, the patient remained disease-free with excellent performance status, demonstrating durable treatment response without evidence of recurrence or metastasis.

The submitted lung tissue from the right upper lobe showed interstitial fibrous tissue hyperplasia, carbon pigment deposition, and lymphocytic infiltration. The bronchial resection margin was negative, with no evidence of carcinoma involvement. Lymph nodes from stations 2, 4, 7, 8, 10, 11, 12, and 13 were all negative for metastatic carcinoma.

## Discussion

Lung cancer is the second most common malignancy worldwide and remains the leading cause of cancer-related mortality (3). NSCLC is the most common histological subtype of lung cancer, accounting for approximately 85% of all lung cancer cases (4). Distant metastasis is the primary driver of mortality in NSCLC. The most frequent sites of distant metastasis in NSCLC are bone, brain, and liver. According to statistics, the proportions of isolated metastases to these organs are 22.3%, 15.4%, and 6.1%, respectively, with corresponding 5-year cancer-specific survival rates of 4.3%, 5.9%, and 2.2% (5).Patients with isolated liver metastasis from NSCLC generally have a poor overall survival. Therefore, exploring more personalized treatment strategies may offer potential survival benefits.

Chemotherapy-based systemic therapy remains the first-line treatment for lung cancer with liver metastases. However, the liver’s unique metabolic microenvironment and immunosuppressive status often lead to suboptimal responses to chemotherapy. Two clinical studies of durvalumab (an anti-PD-L1 antibody) in patients with advanced/metastatic NSCLC demonstrated that baseline liver metastases were associated with shorter survival. While patients derived benefit from immunotherapy monotherapy regardless of PD-L1 expression status, the clinical benefit remained limited (6). The KEYNOTE-189 trial (7) demonstrated that pembrolizumab plus chemotherapy significantly improved overall survival (OS) in patients with baseline liver metastases (12.6 vs. 6.6 months; HR = 0.62, 95% CI: 0.47–0.81; P < 0.001). However, conflicting evidence exists, as some studies reported no statistically significant survival benefit with immune checkpoint inhibitor (ICI)-chemotherapy combinations in hepatic metastasis subgroups (8). The Checkmate 227 trial (9) demonstrated differential efficacy of dual immunotherapy (nivolumab plus ipilimumab) based on metastatic sites: while patients with bone metastases or central nervous system (CNS) involvement derived clinical benefit, those with hepatic metastases showed no significant survival improvement (median OS: 9.5 vs 11.9 months; HR = 1.05, 95% CI: 0.82–1.34; P = 0.68). Combination therapy with antiangiogenic agents and immune checkpoint inhibitors may enhance antitumor efficacy. The IMpower150 trial demonstrated that the atezolizumab plus bevacizumab plus chemotherapy (ABCP) regimen provided significant improvements in both overall survival (OS) and progression-free survival (PFS) compared with bevacizumab plus chemotherapy (BCP) alone. However, this regimen was associated with an increased incidence of grade 3–4 treatment-related adverse events (TRAEs) (10). Pre-immunotherapy radiotherapy (PIRT) has demonstrated potential to improve OS in lung cancer patients with hepatic metastases. However, emerging evidence suggests that administering immune checkpoint inhibitors (ICIs) prior to radiotherapy may suppress antitumor immune responses, potentially leading to inferior clinical outcomes (11). In this case report, the patient achieved sustained disease-free survival (DFS) for 52 months (as of July 2025) following 5 cycles of neoadjuvant chemoimmunotherapy and subsequent surgical resection with confirmed pCR—despite the absence of adjuvant immunotherapy consolidation. However, the necessity of postoperative immunotherapy in pCR patients remains highly controversial. While current evidence supports pCR as a robust prognostic marker for exceptional long-term outcomes in NSCLC, its sufficiency to obviate adjuvant immunotherapy requires further validation through extended follow-up data.

The optimal local treatment modality for liver metastases in lung cancer remains controversial. Hiroyuki Hakoda (12) et al. reported a case of lung cancer with liver metastasis that remained disease-free for 41 months following hepatectomy, demonstrating that resection of an isolated hepatic metastatic lesion from lung cancer may offer the potential for long-term survival. However, contrasting outcomes were observed in two cases of partial hepatectomy for NSCLC liver metastases reported by Ileana et al (13), highlighting the heterogeneity of clinical outcomes in this patient population. Therefore, multidisciplinary team discussion is essential to carefully select patients who may benefit from hepatic surgery. RFA is a safe treatment option for liver metastases from lung cancer, offering acceptable local tumor control, particularly in patients with tumor size ≤3 cm, adenocarcinoma or small cell carcinoma histology, and metachronous liver metastasis (14). Transcatheter arterial chemoembolization (TACE) remains a standard-of-care intervention for hepatic malignancies; however, post-embolization collateral circulation and neoangiogenesis may compromise therapeutic efficacy. For larger hepatic metastases (typically >3 cm), combined modality therapy (TACE plus RFA) has demonstrated superior survival outcomes compared with either modality alone in selected cases. In the present case, the patient achieved durable local control following RFA for hepatic metastases after demonstrating favorable response to prior chemoimmunotherapy, highlighting the potential of sequential locoregional-systemic strategies in multidisciplinary management.

Current evidence supports surgical resection of both primary lung tumors and isolated metastatic lesions in NSCLC patients with oligometastatic disease (e.g., brain/adrenal involvement), provided the primary tumor is non-N2 disease and completely resectable, followed by adjuvant systemic therapy. Conversely, for N2-positive or incompletely resectable primary tumors, systemic therapy remains the mainstay of treatment even if isolated metastases are technically operable. However, no consensus guidelines exist for NSCLC with liver metastases, underscoring the critical need to distinguish true oligometastatic disease when determining therapeutic strategies and prognostic outcomes. While PET-CT and mediastinoscopy serve as essential staging tools, their limited availability in resource-constrained regions may restrict optimal patient selection, potentially affecting treatment efficacy and survival.

## Conclusion

Patients with lung cancer and liver metastases (LCLM) generally exhibit poor prognoses, and surgical resection alone often yields suboptimal outcomes. Consequently, multimodal integrated therapy and personalized treatment strategies have become the standard of care in contemporary practice.

Herein, we present a long-term survivor of oligometastatic NSCLC with liver involvement, who achieved durable disease control through a tailored therapeutic approach incorporating immunotherapy, chemotherapy, surgical resection, and liver-directed local therapy. This case highlights that optimal clinical outcomes for LCLM patients may be attained through MDT-guided decision-making and individualized treatment exploration.

## Data Availability

The original contributions presented in the study are included in the article/**Supplementary Material**. Further inquiries can be directed to the corresponding authors.
