# Supplementary material for: Long-term survival following multimodal therapy for lung cancer with hepatic metastasis: a case report
Source: Front Oncol. 2025 Oct 28;15:1663079. doi: 10.3389/fonc.2025.1663079 (PMC12602229; doi:10.3389/fonc.2025.1663079)
Supplement: Supplementary file 1 [file DataSheet1.docx]

Figure 1


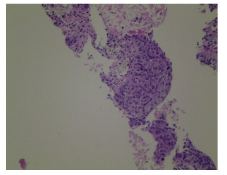
 Pathological examination of CT-guided percutaneous needle biopsy specimens from the pulmonary nodule confirmed squamous cell carcinoma.(2021.2.23)

Figure 2


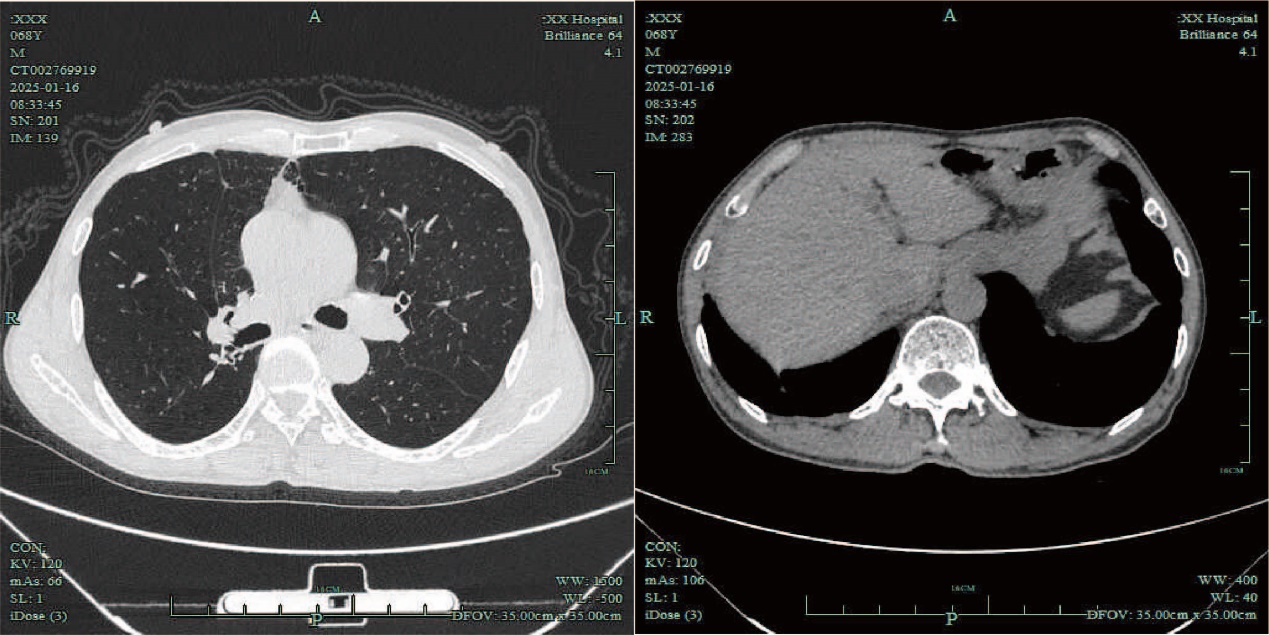


The January 2025 outpatient follow-up chest CT demonstrated no evidence of recurrent pulmonary or hepatic lesions. (2025.1.16)
